# Engineered Vesicles and Hydrogel Technologies for Myocardial Regeneration

**DOI:** 10.3390/gels9100824

**Published:** 2023-10-18

**Authors:** Kaitlyn Ghassemi, Keiko Inouye, Tatevik Takhmazyan, Victor Bonavida, Jia-Wei Yang, Natan Roberto de Barros, Finosh G. Thankam

**Affiliations:** 1 Department of Translational Research, College of Osteopathic Medicine of the Pacific, Western University of Health Sciences, Pomona, CA 91766, USA; kaitlyn.ghassemi@westernu.edu (K.G.); keiko.inouye@westernu.edu (K.I.); tatevik.takhmazyan@westernu.edu (T.T.); victor.bonavida@westernu.edu (V.B.); 2 Terasaki Institute for Biomedical Innovation (TIBI), Los Angeles, CA 90064, USA; jack.yang@terasaki.org (J.-W.Y.); nbarros@terasaki.org (N.R.d.B.)

**Keywords:** engineered vesicles, hydrogel technologies, myocardial regeneration, regenerative cardiology

## Abstract

Increased prevalence of cardiovascular disease and potentially life-threatening complications of myocardial infarction (MI) has led to emerging therapeutic approaches focusing on myocardial regeneration and restoration of physiologic function following infarction. Extracellular vesicle (EV) technology has gained attention owing to the biological potential to modulate cellular immune responses and promote the repair of damaged tissue. Also, EVs are involved in local and distant cellular communication following damage and play an important role in initiating the repair process. Vesicles derived from stem cells and cardiomyocytes (CM) are of particular interest due to their ability to promote cell growth, proliferation, and angiogenesis following MI. Although a promising candidate for myocardial repair, EV technology is limited by the short retention time of vesicles and rapid elimination by the body. There have been several successful attempts to address this shortcoming, which includes hydrogel technology for the sustained bioavailability of EVs. This review discusses and summarizes current understanding regarding EV technology in the context of myocardial repair.

## 1. Introduction

Myocardial Infarction (MI) is highly prevalent and is the leading cause of global mortality. Diverse mechanisms have been proposed utilizing a wide array of molecular signaling pathways and genetic modification aiming at cardiomyocyte (CM) replenishment, as CM loss is the major pathological event associated with MI. MI pathology impairs the physiologic function, CM proliferation, signaling, and phenotypic transformation. Also, MI is associated with inflammatory responses, signaling by microRNAs (miRNAs), long non-coding RNAs (LncRNAs), and fibrotic changes affecting the functioning of CMs. Cell-based therapies have been successfully attempted for cardiac regeneration [1,2].

**Figure 1:**
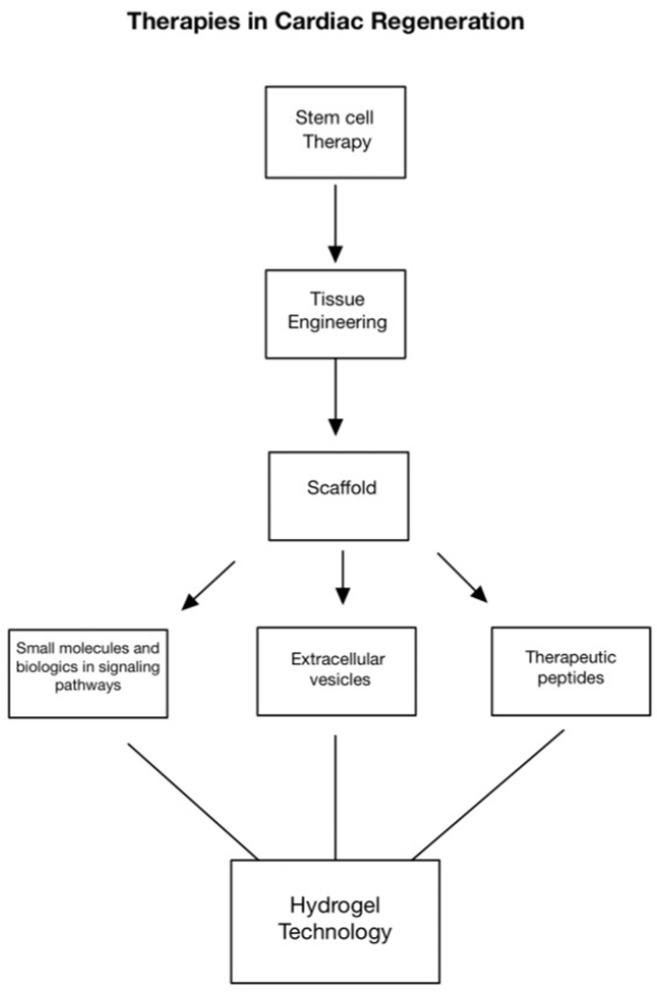
Approaches in cardiac regeneration include stem cell therapy, tissue engineering, scaffolds, altering molecular signaling pathways, extracellular vesicles, and therapeutic peptides. More recently, hydrogel technology has shown to be a promising method for the controlled release of targeted signaling molecules to promote cardiac repair and regeneration.

**Figure 2:**
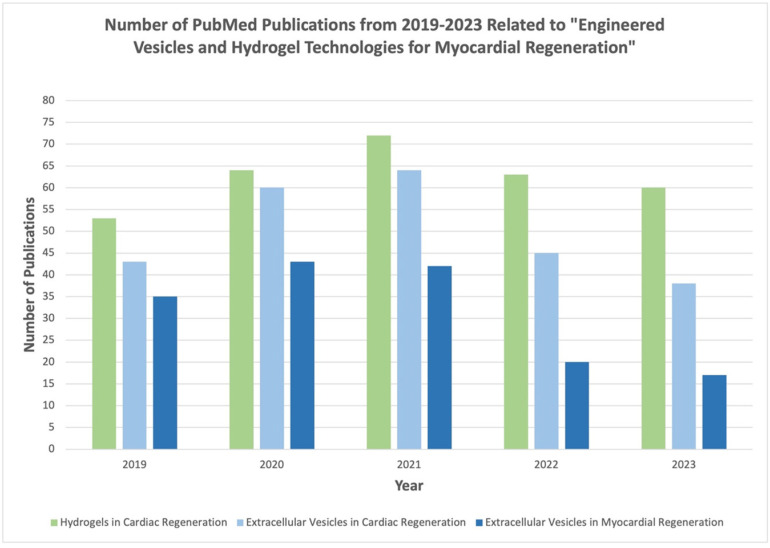
Summary of literature with referenced keywords from 2019–2023 as published in PubMed database.

Interestingly, the regenerative potential of cells largely depends on secretory vesicles or EVs’ cargo (RNAs, proteins, surface receptors, proteins, and transcription factors) carriers distinct from one another. Translationally, EVs can be modified to alter their targets, carrying molecules, and trafficking [3,4]. EV-based communication between neighboring cells is crucial in maintaining integrity and morphology, increasing their potential therapeutic effects. Recent studies unveiled the healing responses of EV-derived communication facilitated by decreased fibrosis, improved cardiac function, reduced oxidative stress, and accelerated myocardial regeneration [4,5,6] (Figure 1). Translationally, the application of EVs requires an ideal delivery system to sustain the therapeutic dose at the infarct zone, which depends on hydrogel systems. Notably, the recent advancements in hydrogel technologies, including phase separation [7], electrospun nanogels [8,9], and polymer technology/engineering [9], promise the next-generation regenerative cardiology. The information regarding hydrogel-based EV delivery to the myocardium is minimal; however, there is an increased trend in the research outcomes along this aspect (Figure 2). This review article focuses on EV engineering and hydrogel technology in myocardial healing following MI.

## 2. Extracellular Vesicles (EVs) in Myocardial Injury and Healing Response

### 2.1. Vesicle-Mediated Cardiac Regeneration

EVs play a key role in cellular communication by delivering these messengers for modulating the effect of functional molecules with physiological effects [10]. Based on etiology and size, EVs are classified into exosomes, apoptotic bodies, and microvesicles. Exosomes are regarded as a ‘*natural drug delivery system (DDS)*’ because they can encapsulate and transport discrete molecules and messengers distinct from their tissue or fluid of origin. EVs have been the target of extensive studies into their potential for restorative therapies [11]. Generally, the EVs have been implicated in mediating cardioprotection through reduced apoptosis and fibrosis and enhanced angiogenesis [10].

Multiple cell and tissue types secrete EVs, which have been detected in bodily fluids such as blood, cerebrospinal fluid (CSF), saliva, and ascites. EVs are involved in both local and distant communication in cardiac tissue following an MI, through which a damaged heart communicates with other tissues and with itself to initiate the repair process [10]. Owing to their endosomal origin, EVs contain membrane transport and fusion proteins (GTPases, annexins, flotillin), tetraspanins (CD9, CD63, CD81, CD82 [FT1]), heat shock proteins (HSC70, HSP90), proteins involved in multivesicular body biogenesis (Alix, TSG101), as well as lipid-related proteins and phospholipases [10,12]. Once released, EVs communicate/interact with surrounding cells or are released into the systemic circulation [13]. This section examines EVs of different cellular and proliferative origins in the context of cardiac regeneration.

Circulatory EVs serve multiple purposes in cardiac regeneration. Firstly, the release of EVs is increased under situations of cellular stress and CM damage, and these vesicles are released into the systemic circulation. Functionally, EVs elicit beneficial responses in self-healing and multidirectional differentiation and regeneration; however, their rapid clearance by the circulatory and cardiac systems with their high level of hemodynamics is challenging [14]. Translationally, the retention of EVs in target tissues has been attributed to the advancements in hydrogel technology, which improved the sustained bioavailability of EVs in the target site. EVs, especially stem cell-derived EVs and other cell types, are considered a promising strategy for improving myocardial function following MI through reducing CM apoptosis, promoting angiogenesis, reducing scar tissue formation and infarct size, and reversing inflammation-induced injury [15].

### 2.2. Immune Cell-Derived EVs

Generally, the inflammatory response in cardiac tissue following MI occurs in two phases. The first phase, the early inflammatory phase, is mediated by M1 macrophages that secrete cytokines and chemokines to remove dead or injured cells through an inflammatory response [4,16,17,18,19]. The second regenerative phase is mediated by M2 macrophages that secrete pro-fibrotic and anti-inflammatory cytokines, including IL-10 and TGF-β [20]. Also, the M2 macrophages promote angiogenesis and extracellular matrix (ECM) deposition through the secretion of VEGF. Dendritic cells (DCs) maintain macrophage homeostasis, and T cells migrate to damaged areas to mediate the inflammatory response [21,22]. Importantly, these cell types secrete EVs to facilitate intercellular and extracellular communication [21,23].

DC-secreted EVs (DC-EVs) bear immune stimulatory molecules. They are composed of complexes of major histocompatibility (MHC)-peptides, T cell co-stimulatory molecules, and molecular elements such as CC-chemokine receptor 7 (CCR7), which guides mature DCs to lymphoid organs such as the spleen to regulate the systemic immune response. These EVs directly activate CD4+ cells in the spleen, which are crucial in improving myocardial healing post-MI [21]. Once activated by DC-EVs, regulatory T cells (Tregs) secrete their own subset of EVs that induce polarization of macrophages into the M2 phenotype to arrest the pro-inflammatory mediators and activate anti-inflammatory mediators. This ultimately results in the suppression of apoptosis of myocardial cells and a reduction in infarct size. The role of B cell-secreted EVs in the context of cardiac ischemia is less clear. However, it is believed to be mediated primarily through activating CD169+ macrophages and MHC-II peptide complexes that induce T cell responses and alter antigen-presenting capacities [24]. Importantly, self-antigens become unintended targets of maladaptive pro-inflammatory immune responses in settings such as cardiac ischemia or coronary artery disease [25]. Macrophage modulation has been known to provide a vital regulatory effect upon damaged myocardium by inducing a pro- or anti-inflammatory state. M1-secreted EVs pro-inflammatory miR-155 into endothelial cells and reduce angiogenesis, exacerbating myocardial injury. In contrast, M2-EVs transport high levels of miR-148 into CM, thus activating the thioredoxin-interacting protein (TXNIP) pathway and inactivating the TLR4/NF-κB/NLRP3 inflammasome signaling pathway, improving the viability of injured CM and a reduction in the size of infarct [21].

### 2.3. Mesenchymal Stem Cells (MSCs)-Derived EVs

MSCs are mesoderm-derived somatic multipotent stem cells originating from bone marrow, umbilical cord, pulp, and fat. In addition to their ability to differentiate into multiple lineages, MSCs have secretory effects that regulate immunosuppressive, anti-inflammatory, pro-angiogenic, or anti-fibrotic responses [26]. These responses are relevant in cardiac regeneration owing to the inherent non-proliferative nature of adult CM being replaced by fibrotic scar tissue, which ultimately accelerates heart failure [27]. The tendency of CMs to undergo apoptosis in response to ischemic stress of damage is inhibited by these MSC-derived EVs, further demonstrating their cardioprotective effects [28].

Several miRNAs have been implicated in the anti-inflammatory processes that MSC-derived EVs promote in cardiomyocytes. For instance, miR-182 has been shown to promote switching M1 macrophages to M2 phenotypes in the peri-infarcted heart tissue. M1 macrophages are recruited to the infarct zone and participate in the inflammation and healing of the myocardium [4,18,19,25]. Importantly, the injection of EVs into damaged myocardium has been shown to reduce the number of CD68+ macrophages, improving the polarization state of macrophages and reducing inflammation [16]. Similarly, miR-233 has been shown to downregulate both SEMA3A and STAT3 expression, reducing the inflammatory response in macrophages [29]. Also, miR-181c has been a key miRNA in EV-mediated T cell regulation, blunting the pro-inflammatory Toll-like receptor 4 (TLR4) pathway [16]. Additionally, miR-19a, miR-22, miR-199a, and miR-214 are involved in EV-mediated anti-apoptotic and anti-oxidative effects of reactive inflammatory endogenous cardiomyocytes post-injury, as seen through the administration of MSC-derived EVs into intramyocardial, intravenous, intracoronary, and intra-pericardiac sac tissues [30]. The pro-angiogenic effects of MSC-derived EVs are mediated by miR-126, miR-210, miR-20a, and VEGF by promoting pro-angiogenic mRNA expression in ischemic tissues [31]. Lastly, the anti-fibrotic effects of MSC-derived EVs are mediated by miR-19a, miR-29, and miR-133 [13,16,29]. Cardiac regeneration and collagen deposition are parallel processes during the repair phase following the ischemic episodes. Scar formation is beneficial for cardiac repair in the short term, as it provides strength and lessens the chances of ventricular rupture; however, it is inversely correlated with cardiac regeneration in the long term [21]. MSC-derived EVs carrying specific miRNAs mediate several ECM proteins and collagen deposition in damaged myocardium [21]. MSC-derived EVs represent a promising therapeutic approach for ameliorating cardiac regeneration and minimizing maladaptive changes in the heart following ischemia.

### 2.4. EVs from Cardiomyocytes (CMs) and Cardiac Fibroblasts (CF)

Healthy CMs have been shown to release EVs containing tropomyosin, myosin-bound protein c, and glyceraldehyde 3-phosphate dehydrogenase (GAPDH). In contrast, CMs exposed to hypoxic conditions secrete various heat shock proteins (HSP27 and HSP90 [13]. On the other hand, the metabolic profile of CFs is altered by EV’s contents, which reduce inflammation and promote angiogenesis [10,11]. There is extensive crosstalk between CMs and the surrounding non-CMs, mostly CFs. Together, these cell types form a structural and functional syncytium. Bidirectional crosstalk mediates mechanical and electrical function in both normal and diseased hearts. The crosstalk mechanism between CMs and CFs has been thought to be due to a combination of paracrine factors, direct cell-cell communication, and interactions in the ECM [31]. Also, the crosstalk between CMs and CFs in ischemic injury has been owed to EVs, mainly due to their ability to transport miRNAs. For instance, miRNAs within EVs change based on systemic and localized disease states, as in the case of CM-derived EVs [32]. Under normal conditions, CMs release EVs containing miRNAs involved in cell growth and survival, such as miR-17, miR-20a, and miR-23b. However, under oxidative and metabolic stress, CMs release EVs containing metabolic regulators such as miR-16, miR-19a, miR-19b, miR-23a, and miR-23b, enhancing angiogenesis and decreasing the uncontrolled collagen deposition in damaged myocardium [33]. Additionally, CM-derived EVs transport miRNAs that contribute to furthering disease states, such as miR-208a [32]. Interestingly, EVs containing miR-208a contributed to fibroblast proliferation and differentiation into myofibroblasts. These cumulative changes increase cardiac stiffness and remodeling, contributing to heart disease progression following ischemic injury [32].

### 2.5. EVs from Cardiosphere-Derived Cells (CDCs)

Several studies have proposed three main sources of new CMs: circulating progenitors from bone marrow that reach the myocardium through systemic circulation, facilitating the differentiation into CMs; pre-existing CMs that divide and multiply through mitosis; and resident/endogenous myocardial multipotent stem cells that proliferate and differentiate into the three main cell types of the heart: CMs, vascular endothelial cells, and smooth muscle cells [34]. Cardiospheres are multicellular clusters that contain several cell types, including CMs, cardiac stem cells (CSCs), cardiac progenitor cells (CPCs), endothelial cells, and smooth muscle cells [35]. Although resident CPCs persist within adult mammalian myocardium, the lack of postnatal mitotic ability limits myocardial regeneration [36]. However, EVs secreted from CDCs promote CM proliferation, enhance angiogenesis, and prevent apoptosis. EVs released by non-injured myocardium in the tissue surrounding the infarct zone contain miRNAs, small molecules, and peptides released at target areas and exert paracrine effects that reprogram CMs and rescue the peri-infarcted region. CDC-derived EVs alter the secretory profile of fibroblasts to anti-fibrotic, anti-apoptotic, and pro-angiogenic and up-regulate the expression of VEGF and stromal-derived factor-1 (SDF-1) and alter miRNA profiles. In addition, CDC-derived EVs share similar mi-RNA profiles to MSC-derived EVs, and increased levels of miR-210, miR-132, and miR-146a-3p promote pro-angiogenic and anti-apoptotic effects in damaged myocardium post-MI [26].

### 2.6. Cardiac Endothelial Cell (CEC)-Derived EVs

Increasing evidence indicated CEC as a key pathologic determinant in cardiac remodeling through interactions with different cells in the myocardium and via secreting autocrine, paracrine, and juxtacrine factors [37]. Certain CEC-derived miRNAs are either up-regulated or downregulated, depending on the type of damage sustained to the myocardium. For example, certain miRNAs such as miRNA-21, miRNA-29a, miRNA-133a, and miRNA-155 were up-regulated in the systemic circulation of patients or experimental models with MI or pressure overload, whereas miRNA-150 was downregulated. In further detail, miRNA-29a was increased in the blood of patients with hypertrophic cardiomyopathy [38]. Serum levels of miRNA-133a, however, were increased in patients with acute MI, unstable angina pectoris, and takotsubo cardiomyopathy [39]. CECs, in contrast to CMs and CFs, have direct contact with circulating blood. Therefore, miRNAs secreted by CECs can act as serum biomarkers for cardiovascular disease [37].

Also, CECs secrete EVs that have a downstream effect on regulating specific B cells. CEC- EVs contain integrin avβ6, which stimulates B cells to release TGF-β. Naïve B cells fail to produce TGF-β without first being stimulated by lipopolysaccharide (LPS). Hence, exposure to EV-secreted avβ6 and LPS generate new immune regulatory cells [40]. Tregs and Bregs are examples of these regulatory immune cells that exert their effects by modulating the effects of specific cells, such as CD4+ effector T cells, that further induce immune inflammation in the body. The downstream is the suppression of effector T cell proliferation, reducing the skewed immune response following ischemic injury to prevent further damage and accelerate healing [21]. In addition, treatment with Tregs reduces inflammation and prevents chronic rejection of heart allografts [40]. The ability of CECs to secrete molecules that regulate immune cells opens new doors to the therapeutic potential of EVs in the context of heart failure and heart transplantation.

### 2.7. Adipose-Derived Stem Cells (ADSC)-Derived EVs

Reperfusion therapy is the typical standard therapy for MI. However, myocardial ischemia/reperfusion (I/R) causes fibrosis and apoptosis, leading to downstream CM injury [6]. Adipose-derived stem cells (ADSCs) have become a preferred cell type for the treatment of I/R injuries as opposed to other MSCs for several reasons: they are relatively easy to harvest, have multilineage differentiation and superior proliferation rate [6,12,41] Luo et al., demonstrated that ADSCs overexpressing miR-126 decreased hypoxic myocardial injury by reducing the expression of inflammation factors. This suggests that ADSC-derived EVs protect myocardial cells from apoptosis, inflammation, and fibrosis, thus preventing myocardial damage and favoring angiogenesis and myocardial repair [42]. Therefore, administering miR-126-enriched EV treatment is a potential therapeutic alternative where stem cell therapy fails to reduce myocardial injury or promote the regeneration process after MI [26].

Lee et al. reported that ADSC-derived conditioned medium (ADSC-CM) was collected and injected into injured cardiac tissue, and cardiac function was examined via echocardiography. In essence, the expression of apoptosis-related proteins, such as p53 up-regulated modulator of apoptosis (PUMA), p-p53, collagen 3, fibronectin, fibrosis-related proteins (ETS-1), and B cell lymphoma 2 (BCL2) was significantly downregulated by ADSC-CM. The mechanism is believed to be due to the presence of miR-221/222, which is present in large amounts in ADSC-CM that target and regulate PUMA and ETS-1 protein levels. The knockdown of PUMA and ETS-1 decreased induction of apoptosis and fibrosis, respectively, through the phosphorylation of p38 and NF-κB mediating apoptosis through the PUMA/p53/BCL2 pathway. The ETS-1/fibronectin/collagen 3 pathway mediated the fibrosis pathway [6]. Overall results showed a protective effect of ADSC-CM from cardiac apoptosis and fibrosis after injury [26,42].

### 2.8. Bone Marrow-Derived Stem Cells (BMSC)-Derived EVs

Bone marrow-derived stem cells (BMSC) repopulate hematopoietic and nonhematopoietic tissues, including endothelium, hepatocytes, neuroectodermal cells, lungs, gut, epithelia, and CMs [43]. Through their angiogenic and anti-inflammatory properties, BMSCs possess regenerative and therapeutic potential for treating cardiac disease [44]. BMSCs differentiate into endothelial progenitor cells (EPCs), angioblasts, or CD34+ cells. These cell types are transplantable into the ischemic myocardium, where they aggregate into foci of neovascularization. This has been demonstrated by two processes: the generation of new blood vessels from vascular endothelial progenitor cells (vasculogenesis) and the formation of new vessels from pre-existing vessels (angiogenesis) [43,45]. Injection of BMSCs directly into damaged tissue promotes the secretion of angiogenic factors such as VEGF, FGF, and Ang-1, further promoting angiogenesis post-injury [45]. Neovascularization benefits cardiac function in the context of previously damaged ischemic myocardium, both anatomically and functionally, and shows great promise for cell therapy in the future [43,44]. Bone marrow-derived hematopoietic stem cells (HSCs) have been studied for their participation in de novo vasculogenesis. However, it is unclear whether their effects are due to paracrine stimulation by surrounding MSCs [46].

Additionally, BMSCs have proven effective in reducing inflammation during the healing responses [45]. The inflammatory process that assists in healing post-MI can have detrimental effects on the tissues if left unchecked. Xu et al. have shown that BMSCs that were pre-conditioned with LPS could mediate post-MI inflammation via the polarization of macrophages toward an anti-inflammatory phenotype [47]. Also, the pre-treated BMSCs secreted diverse cardioprotective growth factors [47]. Myeloid-derived growth factor (Mydgf), secreted by bone marrow-derived monocytes and macrophages, plays an important role in heart regeneration by reducing scar size and improving function after an MI [48]. In a trial by Wang et al., Mydgf was shown to promote CM proliferation post-injury [49].

### 2.9. Epicardial Adipose-Derived Stem Cells-Derived EVs

Epicardial adipose tissue-derived stem cells (EATDS) are a potential mesenchymal stem cell source for cardiac regeneration. The proximity of these cells to the epicardium and their similarities in their vascular supply with cardiac muscle makes them candidates to serve in cardiac regeneration pathways. Studies have shown that EATDS possess a greater capacity to differentiate into cardiomyocytes [50]. While there is much to be understood in the mechanisms and pathways of epicardial fat (EF) and its connections to cardiac-related disease, promising preliminary results elucidated phenotypic changes resulting in cardiac regeneration potential [51].

**Figure 3:**
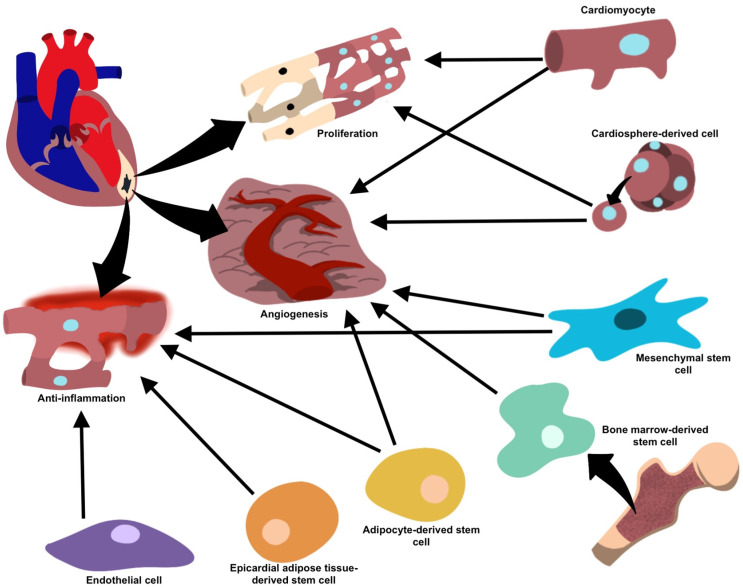
Target cells and their proposed mechanisms for vesicle-mediated cardiac repair.

One study by Lambert et al. showed that EVs secreted by epicardial adipose cells showed higher levels of VEGF and reduced COL18A1, demonstrating the angiogenic potential of these cells [52]. Another study by Ozkaynak et al. observing the myocardial regenerative capacity of EATDS found that these cells demonstrated an increase in vascular density and a clinically significant increase in ejection fraction [50]. Additionally, EATDS appear to play a role in cell differentiation [32]. Yang et al. showed that EVs derived from these cells demonstrated adipogenic capabilities [50]. Exosomal–ribosomal proteins have been investigated for their effectiveness in modulating EATDS, revealing their pro-inflammatory, anti-inflammatory, proliferative, and non-proliferative properties, suggesting their cardiac regeneration potential [51]. Similar to BMSCs, it is unclear whether the reparative properties of these cells are due to the paracrine signaling of neighboring cells [50]. A summary of all the previously discussed cell types and their relationships can be found in Table 1 and Figure 3. Their promise as potential therapeutic targets warrants further investigation.

## 3. Vesicle-Derived Signaling for Cardiac Regeneration

Recent studies have delved into the use of EVs as therapeutic targets. Due to the improved biocompatibility and low immunogenicity compared to cell-based therapies, EVs have become popular candidates for treating cardiac diseases. EVs and their proposed mechanisms of action are summarized in Table 2. MSC-derived EVs (MSC-EVs) are promising candidates for treating a vast array of diseases owing to their regenerative and immunomodulatory properties [41]. Interestingly, MSC-EVs have been shown to protect against myocardial eliciting anti-apoptotic, regenerative, remodeling, anti-inflammatory properties, and neovascularization post-injury [53]. MSC-EVs bear cardioprotective miRNAs (miR-29 and miR-24) along with cardio-offensive (miR-21 and miR-15) as compared to MSCs alone [54]. EVs that contain miR-182-5p, which regulates TLR4 expression, play a role in CM survival following MI via immunomodulatory response [55,56]. Also, the EVs from ADSCs containing miR-93-5p protect against hypoxia-induced autophagy and inflammation, suggesting their healing effects against ischemia [17,57]. Furthermore, the ADSCs treated with miR-146a-containing EVs attenuated acute MI-induced myocardial damage by suppressing the release of pro-inflammatory cytokines [58].

**Figure 4:**
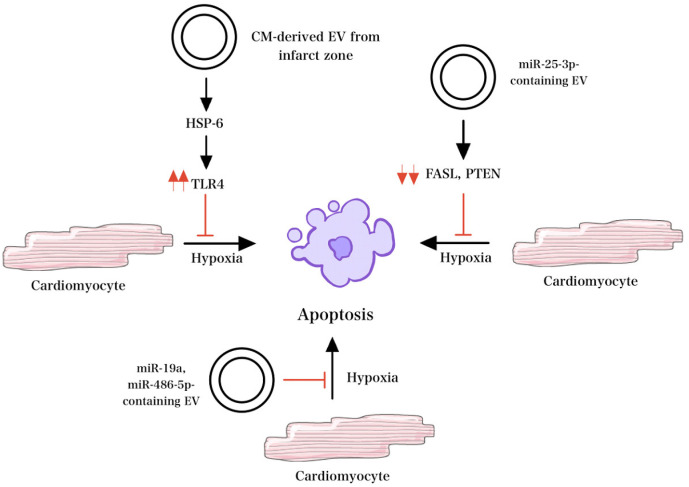
Mechanisms of EV signaling under hypoxic conditions. HSP-6 released from EV in response to hypoxemia activates TLR4 and decreases apoptosis. Similarly, in the setting of hypoxia, miR-25-3p-containing EVs target pro-apoptotic genes FASL and PTEN and inhibit apoptosis. Other EVs that contain miR-19a and miR-486-5p also inhibit apoptosis and increase myocardial repair in the setting of hypoxia.

EVs isolated from hypoxic cells exhibited increased amounts of miR-125b-5p, which improved left ventricular function on delivery into the myocardium immediately post-MI and decreased the infarct size [59]. Additionally, the inhibition of apoptosis was observed following the transfer of miR-19a via MSC-EVs in a rat model [60]. Furthermore, EVs containing miR-25-3p, which targets pro-apoptotic genes FASL and PTEN, and miR-486-5p inhibited apoptosis and increased myocardial repair post-injury, as seen in Figure 4 [61,62]. Also, Atorvastatin-treated MSC-EVs inhibited apoptosis and decreased pro-inflammatory signaling following MI [60].

CM-derived EVs, or “cardiosomes”, are another promising candidate for treating cardiac disease. Cardiosomes isolated from/within the infarct zone release various factors in response to hypoxemia, most notably HSP6, which plays a role in the activation of TLR4 and results in reduced apoptosis [63]. Besides hypoxemia, these cells increase EV secretion under several other stress signals. In addition to protection against apoptosis, cardiosomes promote cell proliferation and angiogenesis when incubated with endothelial cells and improve dysfunctional mitochondria after periods of hypoxia [64]. Cardiosomes extracted from post-ischemic conditioned cells appear to play a role in remodeling repair and protection against reperfusion injury. Morelli et al. found that miR-195, a CM-specific miRNA that aids in the phenoconversion of myofibroblasts, was significantly up-regulated in cardiosomes isolated after MI [65]. Also, HSP70 protein has been shown to up-regulate angiogenesis-related mediators [66]. Cardiosomes containing miR-424-3p, which regulates Ras-related protein signaling, elicit regenerative responses against hypoxia/reoxygenation [67]. In addition, cardiosomes facilitate macrophage polarization, favoring the reparative phenotype and protecting against reperfusion injury [64].

Naïve EVs from cells at the infarct zone have exhibited strong protective and regenerative effects. However, the isolation and manipulation of these EVs have proven difficult, highlighting a need for further investigations [63]. Importantly, cardiosomes serve as valuable biomarkers, aiding in early detection and intervention of MI [64]. In patients with acute MI or other acute coronary syndromes, miR-1 and miR-133a (cardiosome-derived) were significantly elevated [68]. Further research is warranted regarding the therapeutic use of cardiosomes, and their utility as biomarkers is evident.

**Table 2:** List of miRNA targets proposed for EV-mediated cardiac repair and their function.

Animal Model	Target	Function	References
Amphibian and in vitro	miR-1	Promote differentiation into cardiac muscle.	[69]
Amphibian and in vitro	miR-133	Promote myoblast proliferation.	[69]
Mouse	miR-29	Prevents fibrosis by reducing the expression of collagen.	[54]
Mouse	miR-24	Inhibition of cardiomyocyte apoptosis, attenuation of infarct size, reduced cardiac dysfunction.	[54]
Mouse	miR-182-5p	Inhibition of TLR4 activity.	[55,56]
Mouse	miR-212-5p	reduced levels of α-SMA, Collagen I, TGF-β1, and IL-1β.	[22]
Human	miR-93-5p	Promotes proliferation of tumor cells via effects on the PTEN gene.	[70]
Rat	miR-146a	suppressed hypoxic-induced H9c2 injury by suppressing EGR1 and regulating inflammation, apoptosis, and fibrosis by promoting the TLR4/NFκB signaling pathway.	[58]
Human	miR-125b-5p	Suppression of p53 and BAK1 and regulation of apoptosis.	[59]
Rat	miR-19	Prevention of hypoxia/reoxygenation-induced apoptosis and alleviating injury via AKT pathway.	[60]
Mice	miR-25-3p	Targets PTEN and FASL to inhibit apoptosis.	[61]
Rat	miR-486-5p	Suppression of hypoxia/reoxygenation-triggered apoptosis and P13K/AKT pathway activation.	[62]
Human	miR-146	Potentially protective against oxidative stress.	[71]

## 4. Engineered EVs and Hydrogel Technologies for Cardiac Healing

The effectiveness of the therapeutic strategies of EVs has been limited by their biodistribution and off-target effects [35]. However, EVs can be engineered and altered to circumvent these challenges to improve their therapeutic efficacy [72]. Genetically modifying the EV’s surface is a novel approach to improving the targeting capability of therapeutics [72]. By modifying the EV’s surface proteins or adding surface ligands, the interaction of the EV with its target becomes specific [17]. Shortening the time required to attain therapeutic concentration and reducing off-target effects allow maximum therapeutic efficiency [72]. Notably, Ohno et al. demonstrated that cell targeting was enhanced by promoting the expression of GE11 peptide (amino-acid sequence YHWYGYTPQNVI) on EVs, allowing specific binding to epidermal growth factor receptor (EGFR)-expressing breast cancer cells [73]. In another seminal study, muscle and brain targeting peptides were fused to the N terminus of Lamp2b, an EV membrane protein, to deliver short interfering (si) RNA to the brain in mice for a specific gene knockdown [74].

**Figure 5:**
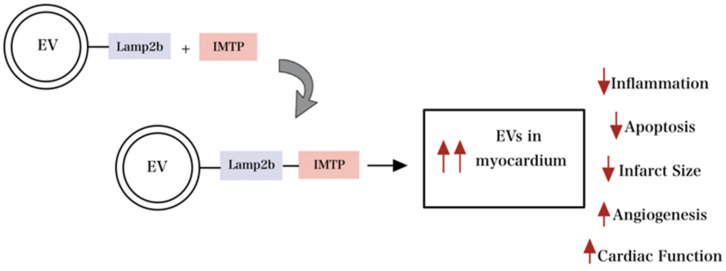
Engineered EV with modification of surface proteins improves targeting capability and maximizes therapeutic efficiency. Engineered EV with modification of surface proteins improves targeting capability and maximizes therapeutic efficiency by reducing inflammation, apoptosis, and infarct size and increasing angiogenesis and overall cardiac function.

Furthermore, Zhu et al. demonstrated that modified EVs with Cyclo (Arg-Gly-Asp-D-Tyr-Lys) (c(RGDyK)) targeting ligand provided excellent Paclitaxel (PTX) drug delivery to glioblastoma cells by crossing the blood-brain barrier [75]. Similarly, EV-mediated signaling during pathogenesis and homeostasis of heart disease in cardiovascular research has been well documented and is a focus for therapeutic delivery [72]. Wang et al. reported that fusing ischemic myocardium-targeting peptide (IMTP) to Lamp2b (IMPT-Lamp2b) resulted in the accumulation of the engineered EVs in the ischemic myocardium, depicted in Figure 5. Thus, through specific targeting, engineered EVs suppressed inflammation and CM apoptosis, promoted angiogenesis, decreased infarct size, and improved cardiac function [17].

Furthermore, engineering EVs to limit their degradation in the endolysosomal pathway has been proposed in several studies. In one example, EVs have been coated with cationic lipids, and pH-sensitive fusogenic peptides disrupt the endolysosomal membrane and lead to the cytosolic release of the EV cargo [76]. In another case, EVs were coated with arginine-rich cell-penetrating peptides to promote EVs’ efficient release and uptake. These modifications allow for the unwanted degradation of EVs in lysosomes, enhancing the EV surface for endolysosomal escape [77].

Another intervention to increase the targeted uptake of EVs has been demonstrated through the bio-material-based sustained release of EVs at an injured site, which includes hydrogels based on hyaluronic acid, alginate, chitosan, collagen, and amphiphilic peptides [77]. Liu et al. demonstrated that a 7 mm hydrogel patch retained ~3 × 10 [10] EVs, ensuring the release for more than seven days following implantation in the heart, improving the activity of injured tissues [70]. This technique of local administration increases cell targeting and decreases unwanted uptake of EVs by neighboring cells.

Moreover, EVs have been demonstrated as delivery vehicles for exogenous mediators such as miRNAs and siRNAs [76]. The most common methods of mRNA loading into EVs include electroporation, transfection, and active loading [78]. Luo et al. concluded that ADSC-EVs transfected with miR-126, a cardioprotective microRNA, showed decreased myocardial injury of the area of infarction, reduced cardiac fibrosis, and inflammatory cytokine expression [42]. Hence, loading RNA into EVs has yielded successful results and shown therapeutic potential for gene therapy for MI.

## 5. EV-Loaded Hydrogels for Cardiac Regeneration

The combination of hydrogels and EVs potentially addresses the obstacles in the myocardial applications of EVs. While EVs exhibit substantial potential in cardiac tissue regeneration due to their versatility and practicality, a critical challenge is their short retention time when utilized in cardiac therapies [79]. Intravenously injected EVs have a short half-life (3 h) and are quickly cleared from the bloodstream due to metabolism, accumulating in the brain, spleen, and liver rather than the heart [80]. Increasing the amount of injected EVs does not extend their in vivo retention. Still, it incites additional complications, such as heightened immune responses and aggravated distribution disparities [35]. Strategies incorporating hydrogels to direct EVs to the heart effectively have been gaining considerable attention [15]. Characterized by their biocompatibility and micro/nanoporous structures, the hydrogels improve EV delivery, prolong their retention at the injection site, and provide structural support and enhanced bioactivity to the surrounding myocardial tissue [81]. Table 3 summarizes various hydrogels recently utilized in EV delivery for cardiac regeneration.

## 6. Natural Hydrogels

Alginates are ionotropic hydrogels proven safe and effective in clinical trials for cardiac tissue preservation, thus being considered an excellent choice for combining with EV therapy [90]. The significant pH sensitivity of alginate hydrogels allows the easy regulation of their mechanical properties and gel structure via ion-exchange reactions, optimizing EV delivery systems. Lv and colleagues combined MSC-derived EVs with alginate hydrogels of varying mechanical properties to evaluate their effect on MI treatment [82]. The study found that gels with 0.5% and 1% calcium chloride solutions released all loaded EVs within ten days. In contrast, those with 2% released 60% of loaded EVs. Moreover, an alginate hydrogel of 1% CaCl_2_ and 2% sodium alginate solution displayed suitable mechanical properties for cardiac tissue engineering applications, with a storage modulus between 400–1800 Pa [82,91]. Another recent research demonstrated that alginate hydrogels extend the retention of DC-derived EVs in the MI area for up to 14 days, offering additional physical support and contributing to post-MI cardiac functional restoration [83]. However, the alginate hydrogels have certain limitations as they are subjected to external environmental factors such as pH, ion concentration, and temperature, which may trigger an uncontrolled dissolution of the gel network, potentially resulting in structural compromise and functional loss [15,92].

Hyaluronic acid (HA) hydrogels, a component of natural ECM, control the stability and mechanical properties through enzymatic crosslinking reactions [93]. HA has been widely used in tissue reconstruction applications, and its safety and effectiveness in cardiac repair have been verified [94]. In a recent study, an injectable HA hydrogel loaded with MSC-derived EVs was engineered [83]. It was delivered to the hearts of mice and pigs through thoracoscopy-guided minimally invasive surgery. The HA hydrogel not only enhanced the retention of EVs in the heart but also prevented pericardial effusion at the injection site [84]. However, in terms of immune cell infiltration, there was no significant difference between the EV-combined HA hydrogel and the injection of HA hydrogel alone. These results suggest additional research to investigate the effects of the combination of EVs and HA hydrogel on immunomodulation [95] and maximize cardiac repair treatments’ efficacy.

Collagen, the most abundant protein in the ECM, forms a three-dimensional fibrous network with crosslinking and porosity that are advantageous for EV transport [96]. A study has exploited this feature of collagen to develop a hydrogel patch capable of delivering EVs for cardiac repair [70]. The research findings suggest that the collagen hydrogel patch continuously releases EVs and remains in the implanted rat infarct zone for at least seven days. Compared to injections, the hydrogel patch maintains most of the cells and microenvironment in the injured area, potentially playing a crucial role in the repair process [70]. Moreover, it was found that EVs isolated from iPSC-cardiomyocytes are enriched with cardiac-specific miRNA, potentially offering better protection for acutely damaged hearts compared to iPSC-derived EVs. However, the implantation of the hydrogel patch typically requires invasive surgery [97,98], so careful consideration is warranted regarding the patient’s condition and surgical risks to balance overall safety and therapeutic outcomes.

Gelatin methacryloyl (GelMA) is a biocompatible material derived from the methacrylation of gelatin and photo-crosslinking using UV or visible light to form porous hydrogels suitable as EV carriers [99]. Recently, a study developed a GelMA spraying technique for non-invasive EV delivery [85]. This method involves mixing GelMA precursors, EVs, and a photoinitiator and spraying them onto the surface of the heart. Then, irradiating with visible light for 30 s, an in situ crosslinked hydrogel patch is formed, enabling local and targeted EV delivery. The results demonstrated that the spraying technique exerts minimal pressure and stress on the surface of the heart, avoiding further tissue damage. Compared to hydrogel patches, the spraying technique can be combined with minimally invasive surgical techniques, reducing the need for open-heart surgery and improving the procedure’s safety [85]. However, to successfully utilize the spraying technique for EV delivery, low-viscosity gel facilitates the spraying process and enables rapid curing, which limits the range of hydrogel material choices [100].

## 7. Composite and Functionalized Hydrogels

In addition to single components, composite hydrogels composed of multiple materials have found widespread application in EV delivery systems [101,102,103]. These composite materials overcome the specific limitations of single materials and offer the advantage of combining multiple effects. For instance, Gil-Cabrerizo et al. developed a hybrid hydrogel by mixing alginate and collagen as an EV carrier for cardiac regeneration [86]. The findings demonstrated that a combination of 1% alginate, 0.5 mg/mL collagen, and 0.25% calcium gluconate hydrogel was ideal for a catheter-based intramyocardial injection system. The hydrogel maintained integrity at the injection site for up to 2 months and sustained the release of EVs for at least seven days. However, the authors pointed out that implementing this hydrogel may pose a risk of cardiac catheter occlusion due to the presence of a pre-cross-linking agent to facilitate hydrogel gelation prior to injection [86].

Another study has shown that adding polyethylene glycol (PEG) improves the performance of cardiac ECM hydrogels, effectively overcoming the limitations of long gelation times and poor physical properties [87]. Cardiac ECM, derived from natural heart tissue, serves as a source of bioactive ECM components, offering the potential to retain more bioactive substances and create an environment necessary for interaction with cardiac cells [104]. Therefore, it has recently been developed as a scaffold for EV delivery [88]. Combining self-assembling peptide hydrogels and EVs into decellularized three-dimensional ECM scaffolds can achieve effective localized therapy and cardiac tissue regeneration following MI. However, the difficulty in obtaining natural heart tissue in sufficient quantities and controlling the quality of hydrogels limit its feasibility in clinical applications [105].

Functionalized hydrogels offer unique potential for targeted EV delivery. These functionalized hydrogels are prepared by introducing specific bioactive substances or adjusting surface properties [106]. For example, a meticulously engineered hydrogel, designed through a combination of phosphoethanolamine and MMP9/H_2_O_2_-responsive PEG, controls EV release under different stages of the inflammatory response in acute myocardial infarction (AMI) [84]. This design strategy facilitates the release of large amounts of EVs when most needed, thereby regulating physiological responses and aiding cardiac recovery.

Moreover, shear-thinning gels composed of adamantane-modified HA and β-cyclodextrin-modified HA are designed to withstand the shear stress exerted during injection [89]. These gels transition to a liquid state during injection for easy delivery within the myocardium. Once they reach the target site and the shear stress is removed, the gels swiftly transition back to a solid structure, optimizing EV delivery. Despite their potential, functionalized hydrogels present practical challenges. The fabrication process is complex, requiring specific equipment and techniques. Moreover, the structure and properties of hydrogels are influenced by various factors such as preparation methods, material characteristics, and environmental conditions [107]. Thus, more research is needed to optimize the fabrication process for improved stability and consistency of these hydrogels.

According to recent research, applying hydrogels for EV delivery offers considerable promise in cardiac tissue regeneration, addressing the inherent challenges of EV treatments. However, numerous opportunities and possibilities remain in integrating EVs with novel hydrogels for cardiac tissue repair. For example, electroconductivity nanocomposite hydrogels embedded with nanoparticles, such as carbon-based materials, polymer nanoparticles, and metal oxide nanocomposites, have demonstrated the ability to enhance cardiac tissue engineering [108,109,110] yet combined with EVs remains relatively uncharted. An electroconductive nanocomposite hydrogel loaded with BMSC-EVs recently has succeeded in spinal cord injury therapy, highlighting the therapeutic potential of incorporating EVs within nanocomposite hydrogels [111]. This research demonstrated that such a combination could effectively guide microglial M2 polarization and promote the regeneration of myelinated axons compared to such hydrogels alone [111]. Thus, a comprehensive investigation of synergistic effects between various hydrogels (stem cell-based, peptide-based, and smart hydrogels) and EVs could provide insights into their complex interactions in cardiac regeneration. Furthermore, it is crucial to tailor hydrogels’ physical and chemical properties and improve in situ crosslinking methods to guarantee stability and controlled release during application. These efforts contribute to developing more effective solutions for myocardial regeneration, improving the quality of life for patients with heart diseases.

## 8. Translational Implications, Limitations and Future

In the context of the heart, mounting evidence suggests that EVs are released from diseased or damaged heart tissue and could be a potential messenger between the heart and other cell types, such as regenerative bone marrow. It would be beneficial to identify further the cell types that initiate signaling following ischemic injury and whether these areas differ quantitatively in the number of EVs secreted based on the extent of the injury. Another potential avenue is to explore the physiologic role of EVs, once released from damaged myocardium, and decipher their role in directly reprogramming local stem cell niches in the myocardium. EVs play a role in transferring molecular messages between different species; however, less is known regarding the therapeutic potential [13]. Extracting and purifying EVs from various tissues and bodily fluids is based on their unique size and density, and purification processes have been crucial for translational applications. Optimizing culture conditions for reproducible EVs without compromising their constitution and therapeutic effects has not been achieved yet [29].

Contrastingly, EVs have demonstrated detrimental effects on cardiac tissue and repair. A seminal study reported that treating healthy cardiomyocytes with Angiotensin 2-treated CF-derived EVs demonstrated extreme hypertrophy [108]. Additionally, another study showed macrophage-derived EVs containing miR-155 promoted inflammation and exacerbated rupture following MI [71]. Moreover, Gallet et al. reported that the body rapidly eliminated injected EVs, which were no longer detectable three hours after myocardial injection [80]. As such, the route of injection impacts the efficacy of EVs. For instance, Monguió-Tortajada et al. demonstrated that systemic injection of EVs led to poor retention and required multiple injections of high EV doses to obtain significant benefits, reducing efficiency and increasing costs [109]. Thus, further investigation into the biodistribution of EVs is warranted for an optimal route of administration. The unknown critical quality attributes (CQAs), which include EV size distribution, pharmacological elements, and thus optimal dosing regimen, limit its use for clinical studies [110]. Additionally, achieving reproducibility of standard EVs by defining complete elements and storage of EVs while preventing loss of function is still being explored to ensure standardization [110]. The biological function of EVs, their application, and subsequent analysis methods warrant further understanding prior to the translation of EVs for the therapy of cardiovascular disease.

Versatile stem cells and their respective derived EVs have been explored in vitro and in vivo, pushing the boundaries of current myocardial regeneration translation research. Many pathways have described the differentiation processes and various regulation modalities to change the downstream effects of these targeted EVs. Regardless of which cell type, EV, target molecule, or delivery method, characterization, and experimental trial warrant improved understanding for the successful clinical application for the regenerative management of surviving myocardium.

## Figures and Tables

**Table 1:** Cell sources and EV contents for cardiac regeneration.

Target Cell	Mechanism Of Repair/Regeneration and Mediators	References
Mesenchymal stem cells	Anti-inflammatory (miR-182, miR-233, miR-181c, miR-19a, miR-22, miR-199a, miR-214), anti-fibrotic (miR-19a, miR-29, miR-133) and pro-angiogenic (miR-126, miR-210, miR-20a, VEGF) processes facilitating repair and regeneration and inhibits the formation of fibrotic scar tissue.	[27]
Cardiomyocytes	Cell growth (miR-17, miR-20a, miR-23b) under normal conditions, and enhanced angiogenesis and decreased collagen deposition (miR-16, miR-19a, miR-19b, miR-23a, miR-23b) under stress.	[33]
Cardiosphere-derived cells	Pro-angiogenic and pro-apoptotic properties (miR-210, miR-132, miR-146a-3p).	[26]
Endothelial cells	Reduce inflammation and facilitate healing (integrin avβ6).	[40]
Adipose-derived stem cells	Anti-inflammatory properties (miR-126) prevent fibrosis and favor angiogenesis, facilitating repair.	[42]
Bone marrow-derived stem cells	Neovascularization and vasculogenesis, once implanted into ischemic cardiac tissue.	[43,44]
Epicardial adipose tissue-derived stem cells	Upregulation in regenerative properties and proliferative/anti-inflammatory proteins during periods of cellular stress or ischemia, as well as differentiation of cell types.	[51]

**Table 3:** Various hydrogels have recently been utilized in EV delivery for cardiac regeneration.

Hydrogel Materials	EV Source	EVs Retention Time (In Vivo)	Application	References
Alginate	MSCs	14 days	Injection	[82]
Alginate	Dendritic cells	14 days	Injection	[83]
Hyaluronic acid (HA)	MSCs	2 days	Injection	[84]
Collagen	iPSCs-derived cardiomyocytes	7 days	Patch	[70]
Gelatin methacryloyl (GelMA)	MSCs	2 days	Spraying	[85]
Alginate and collagen	ADSCs	7 days	Injection	[86]
Cardiac extracellular matrix and polyethylene glycol (PEG)	Cardiospheres	1 day	Injection	[87]
Decellularized cardiac scaffolds and self-assembling peptide hydrogel	MSCs	6 days	3D scaffold	[88]
Distearoyl phosphoethanolamine, polypeptide, and PEG	Regulatory T cells	7 days	injection	[84]
Adamantane and *β*-cyclodextrin modified HA	Endothelial progenitor cells	21 days (in vitro)	injection	[89]

## Data Availability

Not applicable.
